# Total Hip Replacement and Femoral Nail Lengthening for Hip Dysplasia and Limb Length Discrepancy: A Literature Review

**DOI:** 10.7759/cureus.64638

**Published:** 2024-07-16

**Authors:** Vasileios Athanasiou, Spyridon Papagiannis, Panagiotis Antzoulas, Vasileios Papathanidis, Theodoros Stavropoulos, Charalampos Charalampous-Kefalas, Vasileios Bitas

**Affiliations:** 1 Department of Orthopedics and Traumatology, University General Hospital of Patras, Patras, GRC

**Keywords:** total hip arthroplasty, intramedullary distraction, telescopic rod, developmental hip dysplasia, leg length discrepancy

## Abstract

Developmental dysplasia of the hip (DDH) is a serious condition resulting in inadequate acetabular development, distorted bone configuration, and substantially altered hip biomechanics. An extensive leg length discrepancy (LLD) is commonly encountered in such cases, making a total hip arthroplasty (THA) procedure extremely challenging. Although good results in terms of patients’ satisfaction, implant survival rates and overall improved quality of life have been reported, complication rates are considerably higher than primary THA procedures performed for idiopathic osteoarthritis. Reconstructing a dysplastic hip arthrosis and equalizing a preexisting LLD is a technically demanding procedure that is associated with significant bone and soft tissue complications. Intramedullary lengthening through motorized nails has become increasingly popular to address difficult cases with extensive LLD following THA in recent years. However, limited data on femoral lengthening procedures implemented following THA are available considering complications, radiological results, and patient-reported outcomes following staged THA and subsequent femoral lengthening using a femoral magnetically-driven intramedullary lengthening nail. We performed a literature review of the past 10 years in PubMed using the terms neglected hip dislocation, DDH, THA, and intramedullary lengthening nail as keywords. A total amount of eight cases addressing LLD through a telescoping intramedullary nail following THA in DDH have been reported in recent literature. All eight patients underwent primary THA for DDH followed by the implantation of the intramedullary lengthening nail. The mean THA was lengthened by 28.9 mm (from 13.0 to 45.0). The mean time for nail implantation after THA was 11.1 months (from 3.5 to 21). The mean time for lengthening per day through the nail was 0.94 mm (from 0.65 to 1.0) from 26 days to 70 days, and the mean lengthening through the nail was 37.6 mm (from 24.0 to 70.0). Good union and consolidation rates were reported by the authors, while there were no complications. The intramedullary distraction osteogenesis method with a telescopic rod can be an effective method to manage leg length discrepancies while avoiding soft tissue complications in challenging cases of DDH.

## Introduction and background

Developmental dysplasia of the hip (DDH) refers to a wide spectrum of pathologies, including dysplasia of the acetabulum or proximal femur, hip instability, subluxation, or dislocation. It is a serious condition affecting 1 in every 1000 births and is considered among the most common causes of secondary osteoarthritis in adults under the age of 40 years, leading to residual hip pain and osteoarthritis if not properly managed. Total hip arthroplasty (THA) in cases with DDH can be pretty challenging given the diversity of deformities, technical difficulties, and not properly designed prostheses. Leg length discrepancy (LLD), nonunion at the osteotomy site, nerve injuries, postoperative dislocations, and aseptic loosening still remain major postoperative concerns in DDH patients treated with THA. Among postoperative complications, managing potential LLD remains a challenge. Femoral lengthening by means of distraction osteogenesis has been successfully performed using external fixation devices such as the Ilizarov frame or unilateral monorail systems. However, these techniques are technically demanding, not well tolerated by patients and are associated with higher complication rates [1]. To reduce treatment times and complication rates, various hybrid techniques have been developed, including lengthening over an intramedullary nail, lengthening followed by intramedullary nailing and lengthening followed by plating. Leg lengthening in difficult cases with DDH has been revolutionized since the development of motorized intramedullary nails [2-3]. The application of a magnet-operated, remote-controlled intramedullary lengthening nail provided new alternatives to aim for more accurate limb equalization with better functional outcomes and better consolidation rates. The surgeon is able to program and transmit the desired distraction rate and rhythm to the device to achieve the daily distraction goal. These implants are gradually growing in popularity in the management of LLD because of their effectiveness, lower complication rates, better cosmetic results, and patients’ satisfaction rates compared to alternative external device systems. Over the past 10 years, the indications for lengthening nails have increased to include lengthening different bone segments and lengthening in conjunction with the correction of deformities, including demanding cases of LLD in DDH patients following THA [4-5]. We conducted a literature review to evaluate the indications, applications, and clinical results of the femoral nail lengthening technique in cases with severe LLD following THA for DDH.

## Review

The latest 10 years of literature were searched in PubMed, using the terms neglected hip dislocation, DDH, THA, and intramedullary lengthening as key words. To our knowledge, only two articles, including eight cases, have been reported in recently published literature describing the management of LLD following THA in DDH cases using a telescoping intramedullary nail (Table 1). Harkin et al. were the first to publish a case series with this combination, followed by Vogt et al. [6-7]. Patients’ mean age was 30 years (from 17 to 51). The mean LLD was 55.5 mm (from 43.0 to 83). The mean THA was lengthened by 28.9 mm (from 13.0 to 45.0). The mean time for nail implantation after THA was 11.1 months (from 3.5 to 21). The mean time for lengthening per day through the nail was 0.94 mm (from 0.65 to 1.0) from 26 days to 70 days. The mean lengthening through the nail was 37.6 mm (from 24.0 to 70.0). Harkin et al. reported union times of 16, 16, and 24 weeks with excellent Association for the Study and Application of the Method of Ilizarov (ASAMI) scores, whereas Vogt et al. reported consolidation index (days/cm) values of 28.6, 23.5, 25.0, and 24.8. The nail was removed approximately one year after implantation in four patients. The mean residual lengthening was 0.6 mm (from -1 mm to 2 mm). No complications were reported up to the final follow-up at 11 to 27 months. In all cases, a retrograde nail (PRECICE®, NuVasive, San Diego, USA) was used following THA.

**Table 1:** Cases of intramedullary distraction osteogenesis to manage leg-length discrepancies following THA for developmental hip dysplasia n/a: not available; THA: total hip arthroplasty; LLD: leg length discrepancy

Article	Cases	Age/gender	LLD (mm)	Lengthening after THA (mm)	Time of nail implantation (months)	Time of lengthening through the nail (mm/day)	Nail lengthening (mm)	Time to union	Consolidation index (days/cm)	Residual lengthening (mm)	Complications	Follow-up (months)
Harkin et al. [5]	1	40/F	63.5	15.3	21	1.0 /26 days	24.0	16 w	n/a	2.0	No	11
	2	17/F	43.0	18.0	3.5	1.0/30 days	25.0	16 w	n/a	0	No	19.5
	3	28/F	83.0	13.0	8	1.0/70 days	70.0	24 w	n/a	0	No	14
Vogt et al. [6]	4	46/F	60.0	30.0	9	0.97/30-52 days	29.0	n/a	28.6	1	No	18.4 (15-27)
	5	36/M	45.0	35.0	19	0.65/30-52 days	34.0	n/a	23.5	1	No	18.4 (15-27)
	6	51/F	50.0	45.0	10	0.98/30-52 days	43.0	n/a	23.5	2	No	18.4 (15-27)
	7	30/F	50.0	35.0	7	0.97/30-52 days	36.0	n/a	25.0	-1	No	18.4 (15-27)
	8	28/F	50.0	40.0	12	1.0/30-52 days	40.0	n/a	24.8	0	No	18.4 (15-27)

LLD and knee problems, including valgus deformity and discomfort, remain significant concerns in patients with DDH treated with THA [8-9]. Equalizing LLD in DDH patients with high dislocations where the femoral head is dislocated and migrated superiorly and posteriorly with no articulation with any part of the true acetabulum (classified as Hartofilakidis type C) or patients with subluxation over 100% or proximal dislocation over 0.20% of the pelvic height (classified as Crowe type IV) can be challenging [10]. It is noteworthy that Crowe IV DDH patients are more susceptible to postoperative LLD [11]. LLD is defined as mild (less than 1 cm), moderate (between 1 and 2 cm), and severe (greater than 2 cm). LLD can be present in up to 27% of patients who underwent THA for DDH, but only one-third of these patients experience symptoms like walking with a limp, back pain, non-structural scoliosis, and fatigue [12]. Traction neurapraxia of the sciatic nerve is among the most serious considerations while managing excessive LLD following THA [13]. Edwards et al. reported the average lengthening was 2.7 cm (1.9 cm-3.7 cm) in peroneal palsy cases and 4.4 cm (4.0 cm-5.1 cm) in sciatic nerve palsy cases [14]. Schmalzried et al. found that following THA, nerve palsy occurs in about 1% of cases. In about 80% of instances, the sciatic nerve-or its peroneal division-is implicated. According to the extent of the nerve damage, the recovery prognosis varies. Around 41% of the patients recover completely while 44% experience a mild neurological deficit. Around 15% of the patients have a poor outcome characterized by weakness that limits ambulation and/or persistent dysesthesia [15]. According to Brown et al., the incidence ranges from 0.08% to 7.6%. For primary THA, the incidence ranges from 0.09% to 3.7%, and for revision THA from 0% to 7.6% [16]. A maximum acute lengthening of 4 cm can be safely gained during THA with careful monitoring and direct visualization of nerve tension [17-18].

Significant improvements have been made in the field of limb lengthening and reconstruction over the past 10 years [19]. First-generation intramedullary lengthening nails were mechanical nails. Bliskunov, in 1983, was the first to describe intramedullary lengthening nails as a limb-lengthening therapy option, followed by the Albizzia Nail in 1999, and Dean Cole in 2001, who created the internal skeletal kinetic distractor (ISKD) [20-22]. The use of motorized nails for intramedullary lengthening has grown in popularity recently [23]. This technique avoids problems associated with the use of an external fixator, such as patient discomfort or pin tract infections, which raise the risk of bacteremia and osteomyelitis [24-25]. In a recently published review, Barakat et al. reported that the era of the circular frame may be over based on the significant advancement of intramedullary lengthening devices, the lower complication rates, and the higher patients' satisfaction [26]. Second-generation intramedullary lengthening nails are motorized and magnetic. The Fitbone, developed by Baumgart and manufactured by OrthoFix in Lewisville, USA, is the first mechanical, motor-driven intramedullary lengthening nail. An induction coil implanted in the subcutaneous tissue transmits electricity to an electric motor that is part of the implant. The Precice nail is the most recent intramedullary lengthening implant from NuVasive Inc. in San Diego, USA. By adjusting the settings on the external remote controller, the nail can be both distracted and retracted. From the first mechanical nails to motorized nails and, more recently, magnetic lengthening nails, lengthening nails have undergone significant development. However, a number of variables should be taken into account before choosing the right implant [27-30]. Negative prognostic factors for a poor outcome include runaway nails, patients older than 30 years of age, smoking, LLD gain greater than 4 cm, and osteotomy at the same level of previous surgery or trauma. Complications such as pain during lengthening, nerve palsies (most commonly the deep peroneal nerve), device breakage, blockage, runaways, poor regeneration, infection, joint stiffness, and iatrogenic deformity have been reported [31-32]. Powell et al. reported in a recent review study that there is no difference in healing parameters between older (aged 60+) and younger patients, making the minimally invasive limb lengthening and reconstruction (MILN) a safe and practical option for both populations [33].

The indications for lengthening nails have increased to include lengthening of different bone segments and lengthening combined with correction of deformities. A two-stage procedure, starting with a THA construct and followed by limb lengthening using the retrograde intramedullary distraction osteogenesis method with a telescopic rod, has been described to address complex cases of LLD in DDH patients. Despite the low amount of published cases, this recently developed method shows good results in the treatment of DDH with significant LLD, avoiding soft tissue complications. Calder et al. conducted a retrospective comparative study following antegrade and retrograde femoral intramedullary lengthening in 107 patients, both adolescents and adults, and reported excellent results [34]. Recent publications have also reported similar results [35-36].

Preoperative planning is essential in patients undergoing THA for DDH to assess the anatomy and available bone stock, clarify the surgical approach and surgical technique, and determine proper implant choice and positioning [37]. Soft tissue release and balance are necessary to restore the native acetabulum, recover the normal anatomical relationships, and restore the right length [38]. Although the amount of soft tissue release required before implant insertion remains unclear, it is necessary to be as gradual as possible to avoid the risk of palsy and instability. This is useful in equalization of mild LLD, while some authors suggested the use of neuromonitoring to evaluate the sciatic nerve intraoperatively. However, in cases with significant LLD (Hartofilakidis type C or Crowe type IV DDH), the soft tissue release is not sufficient enough to restore proper limb length, which can then be addressed using the intramedullary, femoral lengthening technique. Further studies need to be conducted to confirm the effectiveness and potential complications of this recently suggested technique since our literature review only yielded eight reported cases.

## Conclusions

Treatment of hip dysplasia, especially with extensive LLD, still remains a challenge even for experienced hip surgeons. Reconstructing dysplastic hip arthrosis and equalizing the LLD is a technically demanding surgical procedure that is associated with significant bone and soft tissue complications, such as sciatic nerve palsy. Acute LLD correction during THA is restricted, especially due to sciatic nerve damage. The intramedullary distraction osteogenesis method with a telescopic rod has been recently used to achieve the appropriate leg length while avoiding soft tissue complications. Regular clinical check-ups are required to timely detect and treat any issues that may arise during the lengthening and consolidation periods. The treatment technique itself is laborious and demands tremendous patient compliance. This study shows that the two-stage procedure of THA and the retrograde intramedullary distraction osteogenesis method with a telescopic rod in the treatment of DDH with significant LLD seem to show favorable results. However, the sample of patients is small, and no mid-term or long-term results are available. The intramedullary distraction osteogenesis method with a telescopic rod can be an effective method to manage leg length discrepancies while avoiding soft tissue complications in challenging cases of DDH.
